# Left atrial reservoir strain by speckle-tracking echocardiography predicts prognosis in secondary mitral valve insufficiency

**DOI:** 10.1007/s12471-026-02022-0

**Published:** 2026-02-02

**Authors:** Ricardo Carvalheiro, Miguel Marques Antunes, Vera Vaz Ferreira, Ana Leal, Fernanda Gameiro, Isabel Cardoso, José Viegas, Tânia Mano, Pedro Rio, Sílvia Aguiar Rosa, Ana Teresa Timóteo, Ana Isabel Galrinho, Rui Cruz Ferreira

**Affiliations:** Department of Cardiology, Unidade Local de São José/Hospital de Santa Marta, Lisbon, Portugal

**Keywords:** Mitral Valve Insufficiency, Echocardiography, Speckle Tracking, Prognosis, Heart Failure

## Abstract

**Background:**

Functional mitral regurgitation (FMR) contributes significantly to morbidity and mortality and may result from left ventricular (VFMR) or atrial (AFMR) remodelling. Left atrial reservoir strain (LASR) is a sensitive marker of atrial dysfunction and may offer incremental prognostic value. This study evaluated whether LASR predicts all-cause mortality and heart failure (HF) hospitalizations in FMR, its performance in VFMR versus AFMR, and its utility over standard echocardiographic parameters.

**Methods:**

We retrospectively analyzed 102 patients (mean age 68 ± 14 years, 41.2% female) with at least moderate FMR who underwent transesophageal echocardiography between 2018 and 2023. Patients were categorized into VFMR (LV dysfunction or remodelling) and AFMR (LA enlargement with preserved LV function). LASR was assessed using speckle-tracking echocardiography. Primary and secondary endpoints were all-cause mortality and HF hospitalization, respectively. Cox models evaluated associations with outcomes, including subgroup analysis by LASR quartiles and additional risk stratification combining LASR with peak tricuspid regurgitation (TR) velocity.

**Results:**

LASR was independently associated with all-cause mortality in multivariate Cox regression (adjusted HR = 0.887, *p* = 0.039). Higher LASR quartiles were associated with improved survival (*p* = 0.013). When combined with peak TR velocity in a composite risk model, patients with LASR ≤ 9.0% or TR velocity > 3.0 m/s had significantly higher risks of mortality (HR = 2.853, *p* = 0.012) and HF hospitalization (HR = 3.922, *p* = 0.029).

**Conclusions:**

LASR, particularly when combined with TR velocity, provides strong prognostic value in FMR, supporting its potential role in refining risk assessment.

**Supplementary Information:**

The online version of this article (10.1007/s12471-026-02022-0) contains supplementary material, which is available to authorized users.

## What’s new?

- Left atrial reservoir strain (LASR) is an independent predictor of mortality in secondary or functional mitral regurgitation (FMR), outperforming traditional echocardiographic parameters.
- LASR retains prognostic value across FMR etiologies—both ventriculogenic (VFMR) and atriogenic (AFMR)—and regardless of interventional therapies.
- Combining LASR with peak tricuspid regurgitation velocity effectively identifies high- and low-risk groups for mortality and heart failure hospitalizations.
- These findings support incorporating LASR into routine echocardiographic assessment to enhance risk stratification in SMR.

## Introduction

Functional or secondary mitral regurgitation (FMR) results from LA or LV remodeling that disrupts mitral leaflet coaptation, without intrinsic valve pathology. It is common in heart failure (HF) and associated with increased morbidity and mortality—even among patients with preserved LVEF [1–4]. While therapies like guideline-directed medical treatment and edge-to-edge repair can reduce regurgitation, many patients remain at high risk of adverse outcomes, underscoring the need for better risk stratification [5, 6].

**Fig. 1:**
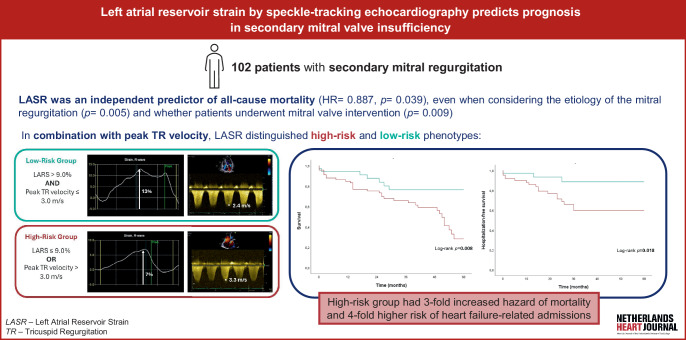
Infographic

Within the broad category of FMR, research has delineated two major pathophysiologic variants: ventricular functional FMR (VFMR), which largely stems from LV remodeling, which displaces the papillary muscles and tethers the mitral leaflets [1, 7], and atrial functional FMR (AFMR), that arises primarily from LA enlargement and mitral annular dilation—often triggered by chronic atrial fibrillation or heart failure with preserved EF—while LV shape and function may be relatively spared [8–10]. Though VFMR has received more clinical attention, AFMR also carries a substantial risk [11].

Left atrial reservoir strain (LASR), assessed via speckle-tracking echocardiography (STE), has emerged as a sensitive marker of LA compliance and early remodelling. Unlike LA volume, LASR can reflect functional decline before structural changes are evident [12]. The significance of LASR has been underscored in various cardiac conditions, particularly in HF, but also in mitral regurgitation syndromes [13, 14]. In MR, reduction in LA strain can appear before overt changes in LA size, possibly capturing the earliest phases of maladaptive remodeling driven by volume overload, rising LV filling pressures, or atrial wall fibrosis [15, 16].

Moreover, clinical data suggest that LASR holds strong prognostic value. Lower LA strain correlates with higher rates of HF hospitalizations and elevated mortality—even after adjusting for conventional metrics such as left atrial volume, LV volumes, and LVEF [17–19]. In both primary and secondary MR, impaired LASR has been associated with adverse outcomes: it predicted clinical events in moderate or asymptomatic MR [20], earlier surgical referral in moderate–severe MR [21], and poor survival following valve repair [17, 22]. Similarly, in secondary MR, LASR independently predicted mortality and HF hospitalizations [18], and it has also been shown to carry prognostic relevance in other structural valve diseases, such as mitral annular calcification [23]. Moreover, in patients undergoing surgical repair of significant MR, LA strain remained independently associated with long-term outcomes [24], and in some studies, improvements in LASR after interventions align with better clinical outcomes [25]. Yet, despite promising insights, the literature remains incomplete regarding how LA reservoir strain compares to or complements established echocardiographic indices in different FMR subtypes.

Most studies focus on single outcomes like mortality, with limited exploration of combined endpoints such as mortality and HF admissions across AFMR and VFMR. This highlights the need to determine whether LASR adds meaningful prognostic value in both subtypes and can improve risk stratification and treatment planning.

This study investigates whether LASR predicts mortality and HF hospitalizations in FMR, assesses its value across etiologies, and evaluates its incremental utility over conventional echocardiographic parameters. The main findings of the present study are summarised in Fig. 1.

## Methods

### Study population

This retrospective study included 102 patients with at least moderate FMR who underwent transesophageal echocardiography (TEE) between January 2018 and June 2023 at *Hospital de Santa Marta—ULS São José, Lisbon, Portugal*. FMR was attributed to either ventricular functional MR (VFMR; left ventricular dysfunction/remodeling) or atrial functional MR (AFMR; isolated left atrial enlargement with preserved LV function). Although patient selection was based on TEE-confirmed moderate-to-severe MR, all echocardiographic analyses were performed using transthoracic echocardiography (TTE) acquired at the time of TEE.

Patients were included if they had at least moderate FMR, defined according to an integrative assessment consistent with guideline-based recommendations for MR severity grading [26]. While an effective regurgitant orifice area (EROA) ≥ 0.20 cm^2^ was typically considered, patients with EROA < 0.20 cm^2^ were also included if a multiparametric evaluation confirmed at least moderate-to-severe MR.

A total of 154 patients were screened. Fifty-two patients (33.8%) were excluded: concomitant severe aortic stenosis (*n* = 11, 7.1%), congenital or hypertrophic cardiomyopathy (*n* = 5, 3.2%), prior mitral valve intervention or the presence of a primary MR component (e.g., prolapse, severe leaflet calcification, cleft) (*n* = 12, 7.8%), inadequate image quality for strain analysis (*n* = 10, 6.5%), and missing clinical data or incomplete follow-up (*n* = 14, 9.1%). The final study cohort consisted of 102 patients (66.2%). The patient selection process is shown in Fig S1.

### Selection process

Patient identification and screening were performed by two investigators (RC, MMA) who independently reviewed echocardiographic reports and images. Discrepancies were resolved by consensus with a senior echocardiographer (AIG).

### Ethics

The study protocol was reviewed and approved by the institutional Ethics Committee of *ULS São José*. Given the retrospective design and the use of de-identified patient data, the requirement for informed consent was waived.

### Echocardiography

TEE was used for diagnosis, but strain and all quantitative analyses were based on TTE. All patients underwent TTE in the left lateral decubitus position using GE Vivid 7 and E9 ultrasound systems (GE-Vingmed Ultrasound, Horten, Norway).

Left ventricular (LV) volumes, ejection fraction (LVEF), left atrial (LA) volumes, and left atrial ejection fraction (LA EF) were measured using the biplane Simpson method and indexed to body surface area, while the left atrioventricular coupling index (LA-LV coupling) was calculated by the ratio of the left atrial end-diastolic volume divided by the left ventricular end-diastolic volume.

Doppler tissue imaging (DTI) was used to assess mitral annular velocities in the apical four-chamber view, with e′ values averaged from septal and lateral annular measurements to determine the E/e′ ratio. Right ventricular systolic function was evaluated using tricuspid annular plane systolic excursion (TAPSE) measured in the focused apical four-chamber view via anatomical M‑mode. Regurgitant volume (RV) and effective regurgitant orifice area (EROA) were assessed using the proximal isovelocity surface area (PISA) method.

Left atrial strain analysis was performed using two-dimensional speckle-tracking echocardiography (2D-STE) on apical four-chamber and two-chamber views. LASR was preferred over contractile or conduit components due to greater reproducibility, especially in atrial fibrillation. Left atrial stiffness index (LA stiffness) was then calculated by dividing the E/e′ ratio by the left atrial reservoir strain (LASR).

### Image quality and reproducibility

LASR analysis required adequate visualization of the left atrial endocardial border in all apical views, acquired at 50–80 frames per second. Recordings were excluded if ≥ 1 segment could not be tracked reliably or if stitching/artifacts precluded strain curve interpretation. Multi-beat averaging was used in cases of atrial fibrillation.

Reproducibility was assessed in a randomly selected subset of 20 patients. Intra-observer variability demonstrated an intraclass correlation coefficient (ICC) of 0.91 with a coefficient of variation (CoV) of 8%, while inter-observer variability yielded an ICC of 0.82 with a CoV of 13%.

### Outcomes and analysis

The primary outcome was all-cause mortality, with heart failure hospitalizations as the secondary outcome. Statistical analyses included standard tests for group comparisons based on variable type and distribution. LASR differences between AFMR and VFMR were assessed using ANCOVA, while Cox regression models evaluated the association of LASR with mortality. Patients were stratified by LASR and peak TR velocity quartiles, and a combined risk model was used to classify high- and low-risk groups. Kaplan-Meier and log-rank tests analyzed survival and hospitalization-free survival, with significance set at *p* < 0.05. Analyses were performed using SPSS v31.

## Results

### Baseline characteristics

Baseline clinical and echocardiographic characteristics are summarized in Table S1. Among 102 patients (mean age 68 ± 14, 41.2% female), common comorbidities included hypertension (63.7%), atrial fibrillation (37.3%), and ischemic heart disease (46.1%). Most patients (60.0%) were NYHA class III or IV. Mean LVEF was 42.6%, and median LASR was 9.0%. Baseline medication use in the study population is presented in Table S2.

Over a median 35-month follow-up (IQR: 16–51), 39.2% of patients died, and 28.4% were hospitalized for HF. A proportion of patients were submitted to mitral valve interventions, including percutaneous repair (26.5%) and surgery (12.7%), with a median time to intervention of 4 months (IQR: 2–10). Differences in outcomes between VFMR and AFMR are presented in Table S3; of note, rates of TEER were significantly higher in VFMR compared with AFMR (*p* = 0.035). At 60 months, patients who underwent mitral valve intervention (surgery or percutaneous repair) showed numerically longer survival compared with those managed without intervention (mean survival 49.3 vs. 40.6 months), although this difference did not reach statistical significance (*p* = 0.214).

AFMR patients were more often female and had higher rates of atrial fibrillation and CKD. VFMR patients had more severe MR by EROA (despite showing no differences in regurgitant volume) and lower LVEF. Despite differences in mitral regurgitation severity, LASR remained non-significantly different between the groups, as determined by an analysis of covariance (ANCOVA) adjusting for effective regurgitant orifice area (EROA) (*p* = 0.116).

### Outcomes and prognostic implications of LASR

Although the groups exhibited differences, Kaplan-Meier analysis showed no significant difference in unadjusted mortality between VFMR and AFMR over 60 months (log-rank χ^2^ = 0.018, = 0.894).

In univariate analysis, lower LASR was significantly associated with increased mortality risk (HR = 0.889, 95% CI: 0.814–0.972, *p* = 0.009). After adjusting for clinical and echocardiographic covariates, LASR remained statistically significant in the multivariate model (HR = 0.887, 95% CI: 0.791–0.994, *p* = 0.039). This association remained significant regardless of MR etiology (HR = 0.872, 95% CI: 0.793–0.960, *p* = 0.005) or whether patients underwent mitral valve intervention (HR = 0.884, 95% CI: 0.806–0.969, *p* = 0.009). Despite lower LASR values in patients with AF compared with those in sinus rhythm (mean LASR 8.9 ± 4.0 vs. 11.4 ± 5.3, *p* = 0.011), the association between LASR and mortality remained significant when analyses were stratified by rhythm (HR = 0.904, 95% CI: 0.828–0.987, *p* = 0.025).

Several other variables were also associated with mortality in univariate analysis, including age (HR = 1.028, *p* = 0.049), peripheral artery disease (HR = 2.542, *p* = 0.031), peak tricuspid regurgitation (TR) velocity (HR = 2.211, *p* = 0.017), and left atrial stiffness (HR = 1.265, *p* = 0.016). However, in the multivariate model, only LASR and peak TR velocity remained significant, with peak TR velocity showing a strong independent association with mortality (HR = 3.196, *p* = 0.009). Left atrial stiffness was excluded from the final model due to collinearity with LASR—prior analysis indicated that when both variables were included, stiffness lost its predictive value (HR = 1.077, *p* = 0.594).

Traditional echocardiographic parameters, such as effective regurgitant orifice area (EROA) (HR = 1.018, *p* = 0.051) and left ventricular end-diastolic volume index (LVTDVi) (HR = 1.007, *p* = 0.092), showed potential associations with mortality in univariate analysis, but failed to reach statistical significance.

The detailed results of the univariate and multivariate analyses are presented in Table S4.

**Fig. 2:**
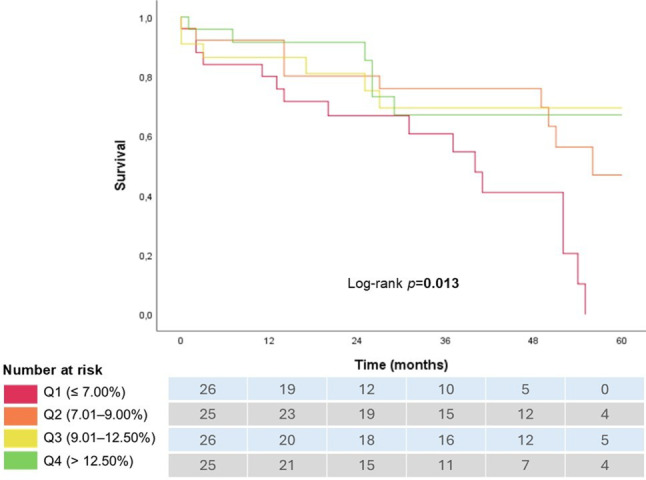
Kaplan–Meier survival curves by LASR quartiles

To further assess the prognostic value of LASR, we conducted a Kaplan-Meier survival analysis stratified by LASR quartiles (Fig. 2): Q1 (≤ 7.00%), Q2 (7.01–9.00%), Q3 (9.01–12.50%), Q4 (> 12.50%). Survival probability differed significantly across quartiles (log-rank χ^2^ = 10.842, *p* = 0.013), with patients in Q1 (LASR ≤ 7.00%) exhibiting the poorest survival outcomes. Higher quartiles were associated with progressively better survival (HR = 0.654, 95% CI: 0.475–0.901, *p* = 0.009).

### Combined effect of LASR and peak TR velocity in prognosis

Given the independent associations of LASR and peak TR velocity with mortality, we assessed their combined predictive value by stratifying peak TR velocity into risk groups: group 1 (≤ 3.0 m/s) and group 2 (> 3.0 m/s).

Patients were then categorized into high-risk and low-risk groups based on LASR and peak TR quartiles: Low-risk: LASR in Q3 or Q4 (> 9.0%) and peak TR velocity in Group 1 (≤ 3.0 m/s). High-risk: LASR in Q1 or Q2 (≤ 9.0%) or peak TR velocity in Group 2 (> 3.0 m/s).

**Fig. 3:**
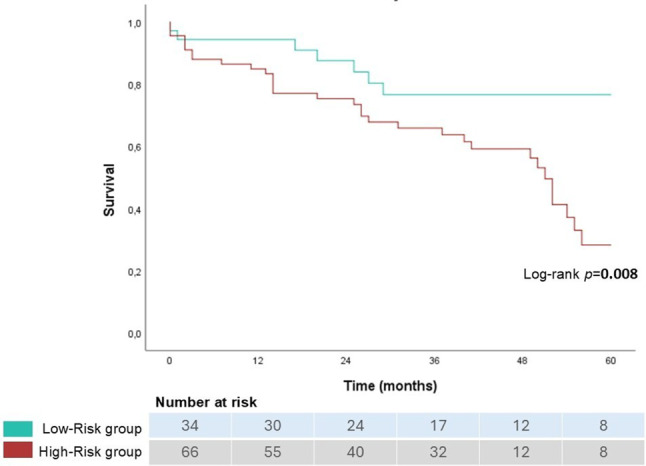
Kaplan–Meier survival curves for high- and low-risk groups based on LASR and peak TR velocity

The incremental value of LASR on top of TR velocity was assessed by constructing combined risk groups. While TR velocity > 3.0 m/s alone identified 33 patients at higher risk, the composite model incorporating LASR reclassified an additional 34 patients into the high-risk group, resulting in 67 patients (65.7% of the cohort) being identified as high risk. Survival probability was significantly lower in the high-risk group (log-rank test: *p* = 0.008), having an almost 3‑fold increased hazard of mortality (HR = 2.853, 95% CI: 1.254–6.489, *p* = 0.012) (Fig. 3).

Beyond mortality risk, the high-risk group was also significantly associated with an increased likelihood of heart failure hospitalizations (Log-rank χ^2^ = 5.563, *p* = 0.018), with high-risk patients having a nearly fourfold higher risk of heart failure-related admissions (HR = 3.922, 95% CI: 1.152–13.355, *p* = 0.029).

## Discussion

In this cohort of patients with moderate to severe functional or secondary mitral regurgitation (FMR), we observed that left atrial reservoir strain (LASR) provided significant prognostic information for all-cause mortality and heart failure hospitalizations. Notably, LASR remained an independent predictor even after adjusting for standard echocardiographic indices and clinical covariates, and its prognostic significance persisted even among patients who underwent mitral valve intervention. These findings align with prior investigations suggesting that LA function, as opposed to mere size or volume, offers superior insight into atrial remodeling and its hemodynamic consequences.

Our results are consonant with human studies showing that impaired LASR predicts adverse outcomes across MR settings: in severe primary MR (post-repair risk), in moderate/asymptomatic disease—including earlier surgical need—and especially in secondary MR where LASR has shown correlations with mortality and HF hospitalizations. We extend this literature by explicitly including VFMR and AFMR populations, confirming LASR’s independent association with mortality after adjustment and intervention status, and demonstrating incremental risk stratification when LASR is integrated with peak TR velocity.

In this study, despite inherent differences in pathophysiology, with VFMR predominantly linked to LV remodeling and AFMR to left atrial enlargement, overall unadjusted mortality did not differ significantly between the two groups. This suggests that once MR severity is established, both VFMR and AFMR can lead to comparable clinical endpoints. Our results indicate that LASR remained an independent prognostic factor even after adjustment for MR etiology, suggesting that its value is not limited to a single pathophysiological subtype. Although AFMR often presents with more atrial fibrillation and relatively preserved LVEF, and VFMR is more commonly associated with ischemic etiology and LV dysfunction, we found LASR to reflect atrial functional reserve, a feature that remained relevant for outcome prediction regardless of the underlying mechanism.

An important observation was that the combined assessment of LASR and peak TR velocity improved risk stratification beyond either parameter alone. Patients categorized as high risk (LASR ≤ 9.0% or peak TR velocity > 3.0 m/s) demonstrated a markedly higher incidence of both mortality and HF admissions. Elevated TR velocity, however, can reflect different pathophysiological states: in some patients, it may primarily represent a reversible hemodynamic consequence of MR-induced pulmonary venous hypertension, which may improve following effective valve intervention; in others, it may indicate more advanced pulmonary vascular disease and right ventricular remodeling, which are associated with limited reversibility and worse outcomes. Accordingly, TR velocity should be interpreted alongside right ventricular function and pulmonary pressures, emphasizing the need for integrated assessment. Prospective studies are warranted to determine its role in guiding patient selection for intervention.

Several clinical implications emerge from these findings. First, LASR may be implemented alongside conventional metrics (e.g., EROA, LV volumes, LVEF) to refine risk stratification, particularly in ambiguous cases where standard indices do not fully capture disease severity or patient-specific vulnerability. Second, our findings highlight that VFMR and AFMR represent distinct patient profiles, and differences in their clinical and echocardiographic characteristics may have implications for future therapeutic approaches. While this hypothesis requires validation in prospective studies, our data emphasize that left atrial function remains a valuable prognostic determinant across both groups. Finally, the additive value of peak TR velocity highlights the interconnectedness of left- and right-sided cardiac function, suggesting a role for comprehensive echocardiographic assessment when evaluating candidates for surgery or transcatheter edge-to-edge repair.

A notable strength of this study is the comprehensive echocardiographic evaluation, including both conventional and speckle-tracking–based parameters, in a population that includes both VFMR and AFMR. In addition, long-term follow-up permitted analysis of both mortality and HF admissions as robust clinical endpoints. However, certain limitations should be acknowledged. This was a retrospective single-center study, which may limit generalizability. Furthermore, the sample size, while sufficient to demonstrate statistical significance, may constrain the depth of certain subgroup analyses. Notably, while LASR remained prognostic irrespective of mitral valve intervention, our retrospective design did not allow assessment of its predictive value for procedural outcomes, which should be addressed in future prospective studies. Future multicenter, prospective investigations will be necessary to confirm these findings, define exact LASR thresholds for clinical decision-making, and explore the impact of targeted treatments on LASR dynamics over time. Finally, detailed characterization of the underlying myocardial substrate (e.g., by cardiac MRI) was not systematically available in this retrospective study. Such phenotyping may provide additional mechanistic insight into the relationship between LASR, myocardial dysfunction, and outcomes, and should be explored in future prospective studies.

## Conclusions

Left atrial reservoir strain was independently associated with mortality and HF hospitalization in patients with moderate to severe secondary mitral regurgitation. In adjusted models, its prognostic value was maintained across both ventricular functional and atrial functional etiologies, underscoring its universal relevance in FMR pathophysiology. Moreover, the combination of LASR and peak tricuspid regurgitation velocity offered enhanced risk discrimination, highlighting the interconnected roles of the left atrium and right heart in driving outcomes. However, its potential therapeutic implications require validation in prospective interventional studies before LASR can be considered in clinical decision-making.

## Supplementary Information

ESM1: Supplementary material 1

ESM2: Supplementary material 2

ESM3: Supplementary material 3

ESM4: Supplementary material 4

ESM5: Supplementary material 5
