# Supplementary figures and images for: Left atrial reservoir strain by speckle-tracking echocardiography predicts prognosis in secondary mitral valve insufficiency
Source: Neth Heart J. 2026 Feb 2;34(3):117–23. doi: 10.1007/s12471-026-02022-0 (PMC12920826; doi:10.1007/s12471-026-02022-0)

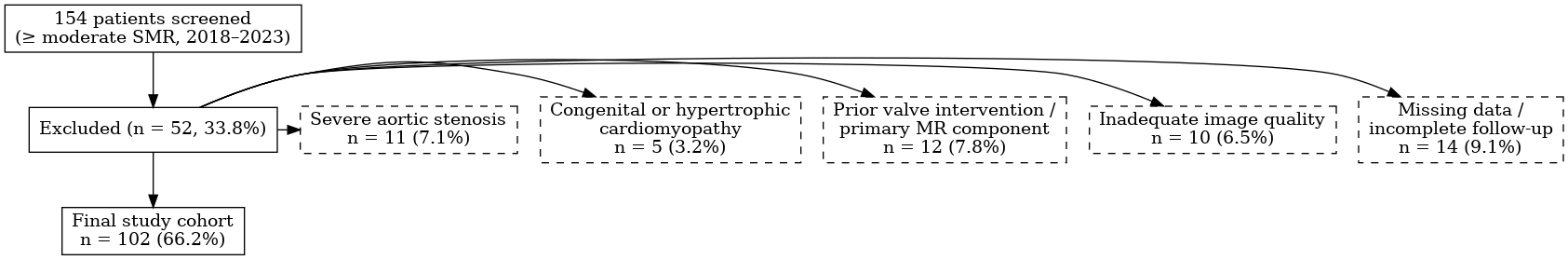

Supplement: Supplementary file 1 — ESM1: Supplementary material 1 [file 12471_2026_2022_MOESM1_ESM.png]
