# Supplementary material for: Left atrial reservoir strain by speckle-tracking echocardiography predicts prognosis in secondary mitral valve insufficiency
Source: Neth Heart J. 2026 Feb 2;34(3):117–23. doi: 10.1007/s12471-026-02022-0 (PMC12920826; doi:10.1007/s12471-026-02022-0)
Supplement: Supplementary file 2 — ESM2: Supplementary material 2 [file 12471_2026_2022_MOESM2_ESM.docx]

|  | **All (n= 102)** | | **Ventricular Functional MR (n=75)** | | **Atrial Functional MR (n=27)** | | ***p*** |
| --- | --- | --- | --- | --- | --- | --- | --- |
| Age in years - mean ± SD | 68 ± 14 | | 66 ± 13 | | 73 ± 15 | | 0.993 |
| Female - n (%) | 42 | (41.2) | 23 | (30.7) | 19 | (70.4) | **<0.001** |
| NYHA class | 2.5 ± 0.8 | | 2.6 ± 0.8 | | 2.3 ± 0.8 | | 0.096 |
| BMI | 26.8 ± 4.7 | | 26.5 ± 4.2 | | 27.9 ± 5.8 | | 0.823 |
| Comorbidities |  |  |  |  |  |  |  |
| Hypertension - n (%) | 65 | (63.7) | 46 | (61.3) | 19 | (70.4) | 0.281 |
| Dyslipidemia - n (%) | 55 | (53.9) | 43 | (57.3) | 12 | (44.4) | 0.324 |
| T2 DM- n (%) | 33 | (32.4) | 28 | (37.3) | 5 | (18.5) | 0.090 |
| Atrial fibrillation/flutter - n (%) | 58 | (56.9) | 37 | (49.3) | 21 | (77.8) | **0.005** |
| OSA/COPD - n (%) | 23 | (22.5) | 18 | (24.0) | 5 | (18.5) | 0.617 |
| Smoking history - n (%) | 22 | (21.5) | 22 | (29.4) | 0 | (0) | **0.008** |
| Chronic Kidney Disease - n (%) | 53 | (52.0) | 44 | (58.7) | 9 | (33.3) | **0.034** |
| Chronic Liver Disease - n (%) | 4 | (3.9) | 4 | (5.3) | 0 | (0) | 0.230 |
| Previous MI - n (%) | 27 | (26.5) | 25 | (33.3) | 2 | (7.4) | **0.011** |
| Coronary Artery Disease - n (%) | 47 | (46.1) | 45 | (60.0) | 2 | (7.4) | **<0.001** |
| Peripheral Artery Disease - n (%) | 11 | (10.8) | 10 | (13.3) | 1 | (3.7) | 0.276 |
| Cerebrovascular Disease - n (%) | 10 | (9.8) | 6 | (8.0) | 4 | (14.8) | 0.277 |
| Active Cancer - n (%) | 7 | (6.9) | 7 | (9.3) | 0 | (0) | 0.249 |
| Laboratory Variables | | | | | | | |
| NTpBNP - median (IQR) | 2798.0 (1279.0-6468.8) | | 3179.0 (1399.0-7509.0) | | 1294.0 (1172.5-3026.0) | | 0.088 |
| Echocardiographic Variables | | | | | | | |
| EROA - mean ± SD | 32.5 ± 17.6 | | 34.8 ± 18.0 | | 25.1 ± 14.1 | | **0.030** |
| Regurgitant Volume - mean ± SD | 47.0 ± 23.8 | | 48.8 ± 23.7 | | 42.5 ± 24.0 | | 0.319 |
| LVTDVi - mean ± SD | 88.2 ± 34.6 | | 97.5 ± 32.6 | | 56.4 ± 17.8 | | **<0.001** |
| LVEF - mean ± SD | 42.6 ± 12.2 | | 37.4 ± 9.2 | | 57.4 ± 5.8 | | **<0.001** |
| LVEF ≥ 40% - n (%) | 62 | (60.8) | 35 | (46.7) | 27 | (100) |  |
| LVEF 30-39% - n (%) | 25 | (24.5) | 25 | (33.3) | 0 | (0) |  |
| LVEF <30% - n (%) | 15 | (14.7) | 15 | (20.0) | 0 | (0) |  |
| TAPSE - mean ± SD | 18.6 ± 4.5 | | 18.1 ± 4.7 | | 19.9 ± 3.9 | | 0.096 |
| Severe tricuspid regurgitation - n (%) | 15 | (14.0) | 8 | (10.7) | 7 | (25.9) | 0.120 |
| Peak TR velocity - mean ± SD | 3.0 ± 0.5 | | 3.0 ± 0.5 | | 3.0 ± 0.6 | | 0.677 |
| LAVi - mean ± SD | 62.5 ± 23.5 | | 62.5 ± 24.0 | | 62.6 ± 22.4 | | 0.987 |
| E velocity - mean ± SD | 106.2 ± 26.6 | | 108.0 ± 26.7 | | 106.2 ± 26.9 | | 0.998 |
| E/A ratio - median (IQR) | 2.0 (1.4-2.9) | | 2.1 (1.4-3.0) | | 1.9 (1.8-2.5) | | 0.869 |
| Deceleration time - mean ± SD | 158.9 ± 44.4 | | 155.2 ± 47.1 | | 171.6 ± 31.6 | | 0.138 |
| E/e' - mean ± SD | 16.0 ± 7.9 | | 17.1 ± 8.1 | | 12.7 ± 6.3 | | **0.037** |
| LASR (%) - median (IQR) | 9.0 (7.0-12.5) | | 9.0 (6.0-12.3) | | 11.0 (8.0-14.0) | | 0.168 |
| LA stiffness - median (IQR) | 1.5 (1.0-2.5) | | 1.6 (1.1-2.8) | | 0.9 (0.7-1.6) | | **0.001** |
| LA-LV coupling - mean ± SD | 0.7 ± 0.4 | | 0.6 ± 0.3 | | 1.0 ± 0.4 | | **<0.001** |
| LAEF (%) - mean ± SD | 21.0 ± 9.4 | | 22.2 ± 8.8 | | 21.3 ± 10.1 | | 0.694 |

**Supplementary Table 1(Table S1) Baseline characteristics**

BMI = Body Mass Index; T2DM = Type 2 Diabetes Mellitus; OSA/COPD = Obstructive Sleep Apnea/Chronic Obstructive Pulmonary Disease; MI = Myocardial Infarction; EROA = Effective Regurgitant Orifice Area; LVTDVi = Indexed Left Ventricular Telediastolic Volume; TAPSE = Tricuspid Annular Plane Systolic Excursion; TR = Tricuspid Regurgitation; LAVi = Indexed Left Atrial Volume; LASR = Left Atrial Reservoir Strain; LA stiffness = Left Atrial Stiffness Index; LA–LV coupling = Left Atrioventricular Coupling Index; LAEF = Left Atrial Ejection Fraction;
