# Supplementary material for: Left atrial reservoir strain by speckle-tracking echocardiography predicts prognosis in secondary mitral valve insufficiency
Source: Neth Heart J. 2026 Feb 2;34(3):117–23. doi: 10.1007/s12471-026-02022-0 (PMC12920826; doi:10.1007/s12471-026-02022-0)
Supplement: Supplementary file 3 — ESM3: Supplementary material 3 [file 12471_2026_2022_MOESM3_ESM.docx]

|  | **All (n= 102)** | | **Ventriculogenic MR (n=75)** | | **Atrial Functional MR (n=27)** | | ***p*** |
| --- | --- | --- | --- | --- | --- | --- | --- |
| Antiplatelet Therapy - n (%) | 48 | (47.1) | 42 | (56.0) | 6 | (22.2) | **0.005** |
| DOAC - n (%) | 54 | (52.9) | 34 | (45.3) | 20 | (74.1) | **0.019** |
| VKA - n (%) | 2 | (2.0) | 1 | (1.3) | 1 | (3.7) | 0.461 |
| ACEi/ARB - n (%) | 33 | (32.4) | 19 | (25.3) | 14 | (51.9) | **0.022** |
| ARNI - n (%) | 46 | (45.1) | 45 | (60.0) | 1 | (3.7) | **<0.001** |
| Beta-Blocker - n (%) | 95 | (93.1) | 73 | (97.3) | 22 | (81.5) | **0.013** |
| SGLT2i - n (%) | 64 | (62.7) | 58 | (77.3) | 6 | (27.3) | **<0.001** |
| MRA - n (%) | 56 | (54.9) | 52 | (69.3) | 4 | (14.8) | **<0.001** |
| Diuretics - n (%) | 85 | (83.3) | 64 | (77.3) | 21 | (77.8) | 0.547 |

***Supplementary Table 2 (Table S2) Baseline Medication Use in the Study Population***

DOAC = Direct Oral Anticoagulant; VKA = Vitamin K Antagonist; ACEi = Angiotensin-Converting Enzyme Inhibitor; ARB = Angiotensin Receptor Blocker; ARNI = Angiotensin Receptor–Neprilysin Inhibitor; SGLT2i = Sodium–Glucose Cotransporter-2 Inhibitor; MRA = Mineralocorticoid Receptor Antagonist.
