# Supplementary material for: Left atrial reservoir strain by speckle-tracking echocardiography predicts prognosis in secondary mitral valve insufficiency
Source: Neth Heart J. 2026 Feb 2;34(3):117–23. doi: 10.1007/s12471-026-02022-0 (PMC12920826; doi:10.1007/s12471-026-02022-0)
Supplement: Supplementary file 4 — ESM4: Supplementary material 4 [file 12471_2026_2022_MOESM4_ESM.docx]

|  | **All (n= 102)** | | **Ventriculogenic MR (n=75)** | | **Atriogenic MR (n=27)** | | ***p*** |
| --- | --- | --- | --- | --- | --- | --- | --- |
| Mortality | 40 | (39.2) | 31 | (41.3) | 9 | (33.3) | 0.465 |
| HF hospitalization | 29 | (28.4) | 22 | (29.3) | 7 | (25.9) | 0.736 |
| TEER | 27 | (26.5) | 24 | (32.0) | 3 | (11.1) | **0.035** |
| Surgery | 13 | (12.7) | 9 | (12.0) | 4 | (14.8) | 0.707 |

**Supplementary Table 3 (Table S3) Outcomes according to functional MR etiology.**

MR = Mitral Regurgitation; HF = Heart Failure; TEER = Transcatheter Edge-to-Edge Repair.
