# Supplementary material for: Left atrial reservoir strain by speckle-tracking echocardiography predicts prognosis in secondary mitral valve insufficiency
Source: Neth Heart J. 2026 Feb 2;34(3):117–23. doi: 10.1007/s12471-026-02022-0 (PMC12920826; doi:10.1007/s12471-026-02022-0)
Supplement: Supplementary file 5 — ESM5: Supplementary material 5 [file 12471_2026_2022_MOESM5_ESM.docx]

|  | Univariable analysis | | Multivariable analysis | |
| --- | --- | --- | --- | --- |
|  | HR (95% CI) | *p* | HR (95% CI) | *p* |
| Age | 1.028 (1.000-1.056) | **0.049** | 1.022 (0.992-1.053) | 0.155 |
| Sex | 1.031 (0.544-1.955) | 0.926 |  |  |
| BMI | 1.042 (0.972-1.116) | 0.247 |  |  |
| Hypertension | 1.240 (0.636-2.417) | 0.528 |  |  |
| Dyslipidemia | 0.909 (0.485-1.706) | 0.767 |  |  |
| T2 DM | 1.291 (0.676-2.465) | 0.436 |  |  |
| Atrial fibrillation/flutter | 1.327 (0.689-2.554) | 0.397 |  |  |
| OSA/COPD | 1.363 (0.678-2.740) | 0.385 |  |  |
| Smoking history | 0.980 (0.616-1.560) | 0.932 |  |  |
| Chronic Kidney Disease | 1.643 (0.854-3.161) | 0.137 |  |  |
| Chronic Liver Disease | 1.387 (0.330-5.825) | 0.655 |  |  |
| Coronary Artery Disease | 1.385 (0.738-2.599) | 0.311 |  |  |
| Peripheral Artery Disease | 2.459 (1.065-5.674) | **0.035** | 3.285 (0.971-11.111) | 0.560 |
| Cerebrovascular Disease | 1.654 (0.642-4.263) | 0.297 |  |  |
| Active Cancer | 1.060 (0.510-2.201) | 0.876 |  |  |
| EROA | 1.018 (1.000-1.036) | 0.051 |  |  |
| Regurgitant Volume | 1.011 (0.997-1.026) | 0.125 |  |  |
| LVTDVi | 1.008 (0.999-1.017) | 0.092 |  |  |
| LVEF | 0.986 (0.960-1.012) | 0.283 |  |  |
| TAPSE | 0.951 (0.886-1.022) | 0.170 |  |  |
| Tricuspid regurgitation grade | 1.118 (0.781-1.602) | 0.542 |  |  |
| Peak TR velocity | 2.042 (1.059-3.939) | **0.033** | 3.196 (1.343-7.604) | **0.009** |
| LAVi | 1.010 (0.997-1.022) | 0.134 |  |  |
| E velocity | 1.006 (0.993-1.020) | 0.343 |  |  |
| E/A ratio | 1.136 (0.801-1.613) | 0.474 |  |  |
| Deceleration time | 0.997 (0.990-1.004) | 0.417 |  |  |
| E/e' | 1.018 (0.978-1.060) | 0.384 |  |  |
| LASR | 0.889 (0.815-0.972) | **0.009** | 0.887 (0.791-0.994) | **0.039** |
| LA stiffness | 1.265 (1.045-1.530) | 0.016 |  |  |
| LA-LV coupling | 0.802 (0.277-2.326) | 0.685 |  |  |
| LAEF | 1.691 (0.058-49.237) | 0.760 |  |  |

**Supplementary Table 4 (Table S4)** **Univariable and multivariable Cox regression analysis for predictors of mortality.**
HR = hazard ratio; CI = confidence interval; p < 0.05 considered statistically significant

BMI = Body Mass Index; T2 DM = Type 2 Diabetes Mellitus; OSA/COPD = Obstructive Sleep Apnea/Chronic Obstructive Pulmonary Disease; MI = Myocardial Infarction ; EROA = Effective Regurgitant Orifice Area; LVTDVi = Indexed Left Ventricular Telediastolic Volume; TAPSE = Tricuspid Annular Plane Systolic Excursion; TR = Tricuspid Regurgitation; LAVi = Indexed Left Atrial Volume; LASR = Left Atrial Reservoir Strain; LA stiffness = Left Atrial Stiffness Index; LA-LV coupling = Left Atrioventricular Coupling Index; LAEF = Left Atrial Ejection Fractrion
